# The lyophilized chloroplasts store synthetic DARPin G3 as bioactive encapsulated organelles

**DOI:** 10.1186/s13036-023-00383-3

**Published:** 2023-10-05

**Authors:** Maryam Ehsasatvatan, Bahram Baghban Kohnehrouz

**Affiliations:** Department of Plant Breeding & Biotechnology, Faculty of Agriculture, University of Tabriz, Tabriz, 51666 Iran

**Keywords:** Chloroplast transformation, DARPin, Lyophilization, Molecular farming, Protein stability, Tobacco, Transplastomic plant

## Abstract

**Background:**

The high cost of fermentation, purification, cold storage and transportation, short shelf life, and sterile delivery methods of biopharmaceuticals, is a matter for producers and consumers as well. Since the FDA has now approved plant cells for large-scale, cost-effective biopharmaceutical production, the isolation and lyophilization of transplastomic chloroplasts can cover concerns about limitations. DARPins are engineered small single-domain proteins that have been selected to bind to HER2 with high affinity and specificity. HER2 is an oncogene involved in abnormal cell growth in some cancers and the target molecule for cancer immunotherapy.

**Results:**

In this study, we reported the prolonged stability and functionality of DARPin G3 in lyophilized transplastomic tobacco leaves and chloroplasts. Western blot analysis of lyophilized leaves and chloroplasts stored at room temperature for up to nine months showed that the DARPin G3 protein was stable and preserved proper folding. Lyophilization of leaves and isolated chloroplasts increased DARPin G3 protein concentrations by 16 and 32-fold, respectively. The HER2-binding assay demonstrated that the chloroplast-made DARPin G3 can maintain its stability and binding activity without any affinity drop in lyophilized leaf materials throughout this study for more than nine months at room temperature.

**Conclusion:**

Lyophilization of chloroplasts expressing DARPin G3 would further reduce costs and simplify downstream processing, purification, and storage. Compressed packages of lyophilized chloroplasts were much more effective than lyophilized transplastomic leaves considering occupied space and downstream extraction and purification of DARPin G3 after nine months. These methods facilitate any relevant formulation practices for these compounds to meet any demand-oriented needs.

## Background

The biological production of bioactive designed proteins in bulk quantities is one of the methods to meet the growing needs in the protein-related market and industries. Reducing production costs in mass production systems is a major challenge for all manufacturing companies. Among the recombinant protein production platforms, transplastomic plant-made pharmaceuticals are highly demanded and can be one of the most cost-effective routes for meeting the needs of rapidly growing recombinant drug companies. Although the currently used production systems based on mammalian, insect, yeast, or bacterial cell cultures have been developed and improved in accordance with current good manufacturing practice [1], plant-based protocols are facing some ups and downs in the commercialization process. Here we examined the potential of lyophilization in the long-term storage of centrifugally compressed isolated chloroplasts as well as intact leaves by developing transplastomic tobacco plants expressing the antibody mimetic DARPin G3. It is assumed that the longevity and low-cost preservation methods remove any negative speculation on the efficiency of plant-based protocols in the formulation of any medicinal recipes. Typically, focusing on a small number of platforms makes meeting the unique requirements of certain target proteins difficult; this is especially true for recombinant proteins that are needed in small quantities for individual patients as well as large quantities for mass production or where rapid scale-up is required [2].

Compared to bacterial, yeast, and mammalian cell culture systems, the production of pharmaceutical proteins in plants offers several advantages, including low cost, a high production rate, easy scale-up with relatively low demand and a simplified procedure, and importantly, a low risk of product contamination by human or animal pathogens and endotoxins, resulting in increased patient safety [3–5]. Additionally, plants are able to perform appropriate eukaryotic protein post-translational modifications such as glycosylation, disulfide bond formation, folding, and multimeric assembly, which are frequently crucial for the biological activity and stability of many mammalian proteins [5–7].

Although stable nuclear transformation is useful for variety research, it is inefficient for protein accumulation, leading to low expression levels that have hampered the production of biopharmaceuticals at industrial levels in plants as compared to those produced by other methods [8, 9]. Instead, plastidial biopharming as a highly efficient bioreactor offers several unique advantages over nuclear transformation, which make the system ideal for plant-made biopharmaceuticals. This includes multiple genes transferring into the chloroplast genome in a single transformation event; high-level expression of transgenes due to high copy numbers of the plastome in each plant cell (several thousands); a lack of gene silencing and positional effects due to site-specific integration, which minimizes the number of events required for screening; and the absence of epigenetic effects and transgene containment due to maternal inheritance of plastids [10].

Transplastomic leaf lyophilization offers additional advantages that further reduce costs and facilitate processing, purification, storage, and immunization. Furthermore, this method increases the concentration of the recombinant products, making them more stable (retaining appropriate folding, disulfide bond formation, and functional efficacy) even after long-term room-temperature storage, eliminating the need for costly cold chains and simplifying transportation [11].

Antibody mimetics, which are small, designed amino acid sequences to mimic structural features of antibody complementarity-determining regions, have inhibitory properties similar to mAbs [12]. These antibody substitutes have a completely different protein topology, which provides advantages such as single-chain buildup (enabling the simple production and subsequent construction of fusion proteins); small size (for good tissue penetration); and, in many cases, high thermostability (enabling long-term storage at room temperature without loss of activity) [13]. Designed ankyrin repeat proteins (DARPins) are one of several antibody mimetics with binding functions that have been developed in recent years [14].

Human epidermal growth factor receptor-2 (HER2) has tyrosine kinase activity that has been linked to the growth of malignancies of various origins and is a validated target for monoclonal antibodies and kinase inhibitors [15, 16]. Utilizing a new approach with DARPins as alternative binders can cause stronger cytotoxic effects on the HER2-addicted breast cancer cell lines [17, 18]. HER2 is overexpressed in approximately 20–30% of breast cancer tumors (Yan et al., 2015). Trastuzumab and Pertuzumab, two currently available monoclonal antibodies and tyrosine kinase inhibitor drugs, rarely achieve complete disease control (Escrivá-de-Romaní et al., 2018). However, the production of antibodies is comparatively expensive and challenging. Additionally, the massive size of immunoglobulin G is rather large for therapeutic applications that require efficient tissue penetration (Binz et al., 2003). DARPin G3, with a low molecular weight (14–15 kDa) and picomolar affinity for HER2 (91 pmol/L), can be used as a molecular imaging agent to visualize HER2-positive tumors (Jost et al., 2013; Zahnd et al., 2007; Zahnd et al., 2010).

We recently and for the first time developed tobacco transplastomic lines producing high levels of DARPin G3 as an antibody mimetic for HER2 targeting in HER2-positive cancers [19]. Our previous study’s results demonstrated that the DARPin G3 produced in chloroplasts binds HER2 with high affinity, as previously reported for this DARPin G3 expressed in *E. coli* [20] or *P. pastoris* [17], as shown using an enzyme-linked immunosorbent assay (ELISA). The HER2-specific binding of chloroplast-made DARPin G3 on the cancer cell membrane was revealed by flow cytometry and immunofluorescent microscopy.

The present research was designed and conducted to evaluate the bioactivity and longevity of bio-encapsulated chloroplast-made DARPin G3 from lyophilized intact chloroplasts and leaves. Our findings show that even after nine months of room-temperature storage, the chloroplast-made DARPin G3 protein remained stable and could be detected at high levels in lyophilized chloroplasts and leaves. An enzyme-linked immunosorbent assay confirmed the binding affinity of chloroplast-made DARPin G3, purified from freeze-dried leaf materials, to HER2 receptors. Immunofluorescent microscopy revealed the functionality of the HER2-specific binding of chloroplast-made DARPin G3 on the adenocarcinoma cell surface. According to these results, DARPin G3 preservation in lyophilized leaf materials, particularly in lyophilized chloroplasts, would simplify processing, purification, and storage, lowering costs and enabling the practical production of scaffold proteins in chloroplast-based expression systems.

## Results

### Chloroplast isolation

**Fig. 1:**
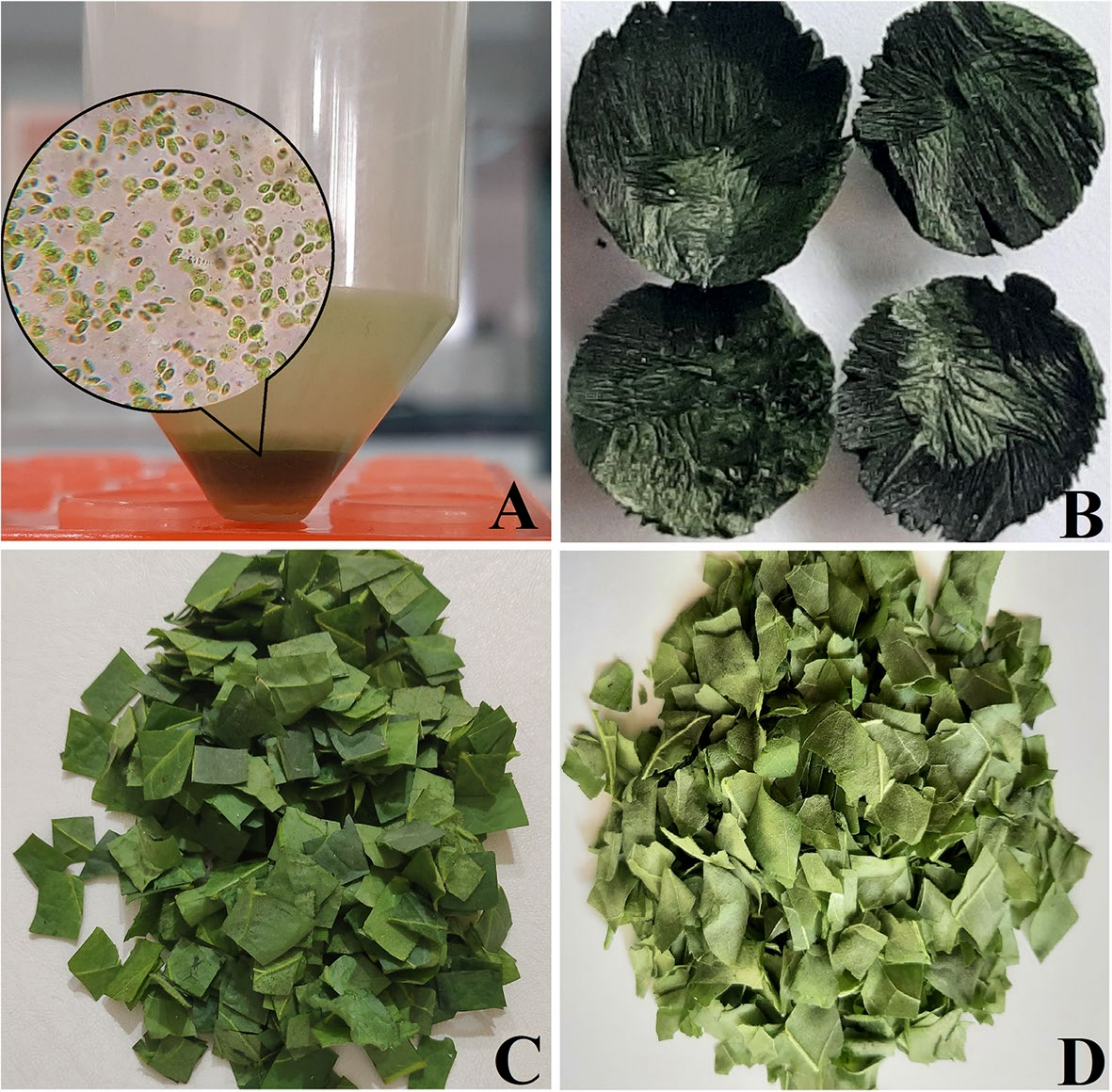
Preparation of lyophilized powdered tobacco leaves and chloroplasts expressing the DARPin G3 protein. **A** freshly extracted chloroplasts along with a microscopic image of them; (**B**) lyophilized chloroplasts; (**C**) fresh leaves; and (**D**) lyophilized leaves

Isolation of chloroplasts was performed for comparative analysis of the DARPin G3 content in fresh and lyophilized chloroplasts and intact leaves. The purity and integrity of the isolated chloroplasts were examined and confirmed using optical microscopy, showing the above 99% intact chloroplasts in the sample (Fig. 1A). Based on the volumetric calculation of a single typical chloroplast (3 μm in diameter) and photosynthetic parenchyma cell (40–80 μm width and length), considering 100–200 chloroplasts per cell, the chloroplast content of each cell was estimated at around 3.4–6.8%. The volumetric estimation is an acceptable approximation for the isolated chloroplasts content of 6.38%, which is achieved by mass estimation based on the fresh weight of leaves and isolated chloroplasts. The isolated chloroplasts were used for protein extraction in subsequent analyses or for lyophilization.

### Lyophilization

Lyophilization of DARPin G3-expressing tobacco leaves and isolated chloroplasts was performed to facilitate long-term storage at room temperature for the subsequent purification and evaluation of DARPin G3 stability and activity (Fig. 1B-D). We observed approximately a 93% and 48% decrease in weight due to the removal of water content in lyophilized leaves and lyophilized chloroplastic pellets, respectively.

### DARPin G3 quantitation

**Fig. 2:**
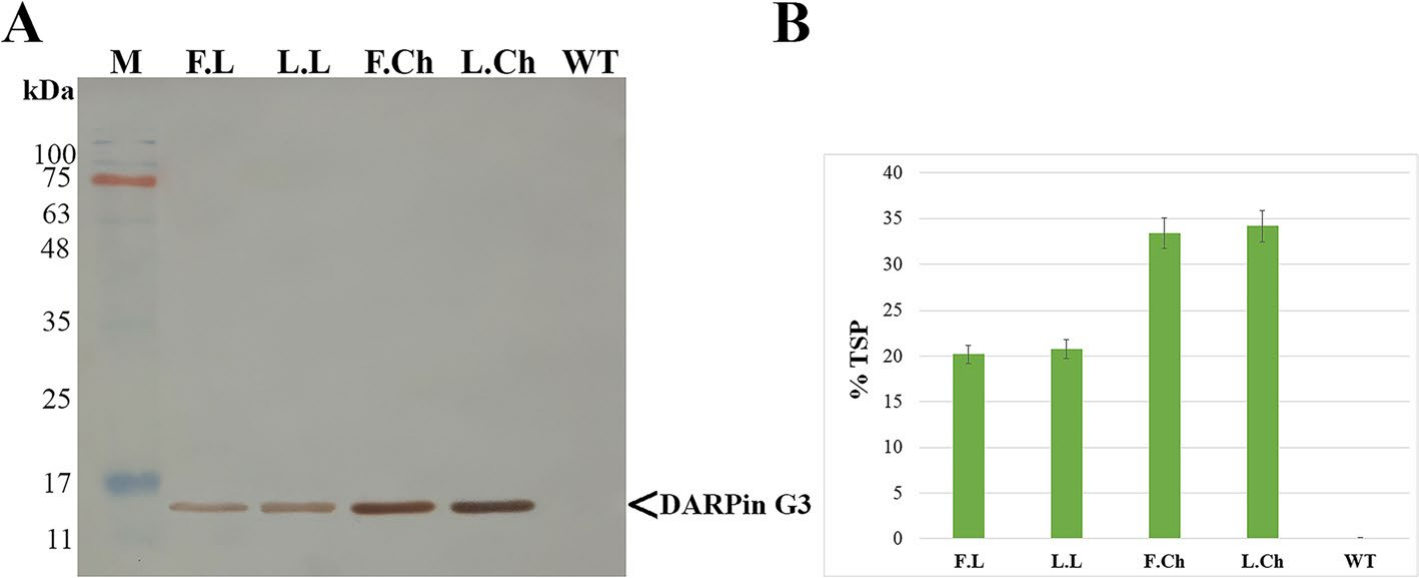
Accumulation and quantification of DARPin G3 in fresh and lyophilized materials. **A** Immunoblot of DARPin G3 protein in fresh (F) and lyophilized (L) tobacco leaves (L) and chloroplasts (Ch). TSP extracted from a mature leaf of wild-type (WT) was also loaded as a control. A normalized amount of fresh and lyophilized leaves and chloroplasts was used for protein extraction, and an equal amount (50 µg) of protein extract was loaded. DARPin G3 and molecular weight marker (Broad Range Protein Molecular Weight Markers) positions are indicated on the margins. **B** ELISA analysis for the chloroplast-made DARPin G3 content in fresh and lyophilized materials. The total soluble protein of the wild-type (WT) tobacco plant was used as a negative control. Data are the means ± SD of three independent experiments. Error bars represent the standard deviation of the mean

DARPin G3 accumulation, integrity, and stability in the fresh and lyophilized leaves and chloroplasts of transplastomic plants after nine months of storage at room temperature were investigated by Western blot analysis. A Western blot was performed with cellular and chloroplastic protein extracts from fresh and lyophilized materials of transplastomic plants. As the N-terminal of DARPin G3 is equipped with a histidine-glutamate (HE)_3_-tag, DARPin G3 was probed with a rabbit anti-His-tag antibody as the primary antibody and an HRP-conjugated goat anti-rabbit antibody as the secondary antibody on SDS-PAGE under reducing conditions. The DARPin G3 protein with an accurate molecular mass of ~ 15 kDa was detected in lyophilized chloroplasts and leaves of transplastomic plants (Fig. 2A). Western blots showed an apparent difference in DARPin G3 intensity between equal protein loading (50 µg) from fresh and lyophilized leaves against fresh and lyophilized chloroplasts. In addition to the higher number of chloroplasts per cell and the higher polyploidy level of each chloroplast, higher DARPin G3 concentrations and amounts in chloroplastic samples are consistent with the fact that chloroplast proteins comprise 40% of total cellular protein [21].

The quantitation of the chloroplast-made DARPin G3 in fresh and lyophilized leaves and chloroplasts of tobacco transplastomic plants was performed by ELISA. The expressed amounts of chloroplast-made DARPin G3 protein accounted for approximately 20% of TSP in both fresh and lyophilized leaves of transplastomic plants. On this basis, the production yield of DARPin G3 was estimated to be 3.4 mg/g of fresh leaf weight and 55.2 mg/g of lyophilized leaf weight in the transplastomic plants. The DARPin G3 protein content in fresh and lyophilized chloroplasts was estimated to be approximately 33% of TSP, which is representative of 56.1 mg/g fresh chloroplast weight and 111.3 mg/g lyophilized chloroplasts weight (Fig. 2B). Therefore, the content of DARPin G3 increased 16- and 32-fold in lyophilized leaves and chloroplasts when compared to fresh leaves, respectively. The ELISA approach proved transgene expression without imposing a yield penalty. In addition, the significant difference in the accumulation of DARPin G3 protein in intact cells and chloroplasts further confirms the cellular/chloroplastic protein ratio again.

These analyses revealed that chloroplast-made DARPin G3 remained soluble and stable even after lyophilization and storage for at least nine months at an ambient temperature comparable to their respective total extracts from fresh leaves and chloroplasts.

### Purification of chloroplast-made DARPin G3

**Fig. 3:**
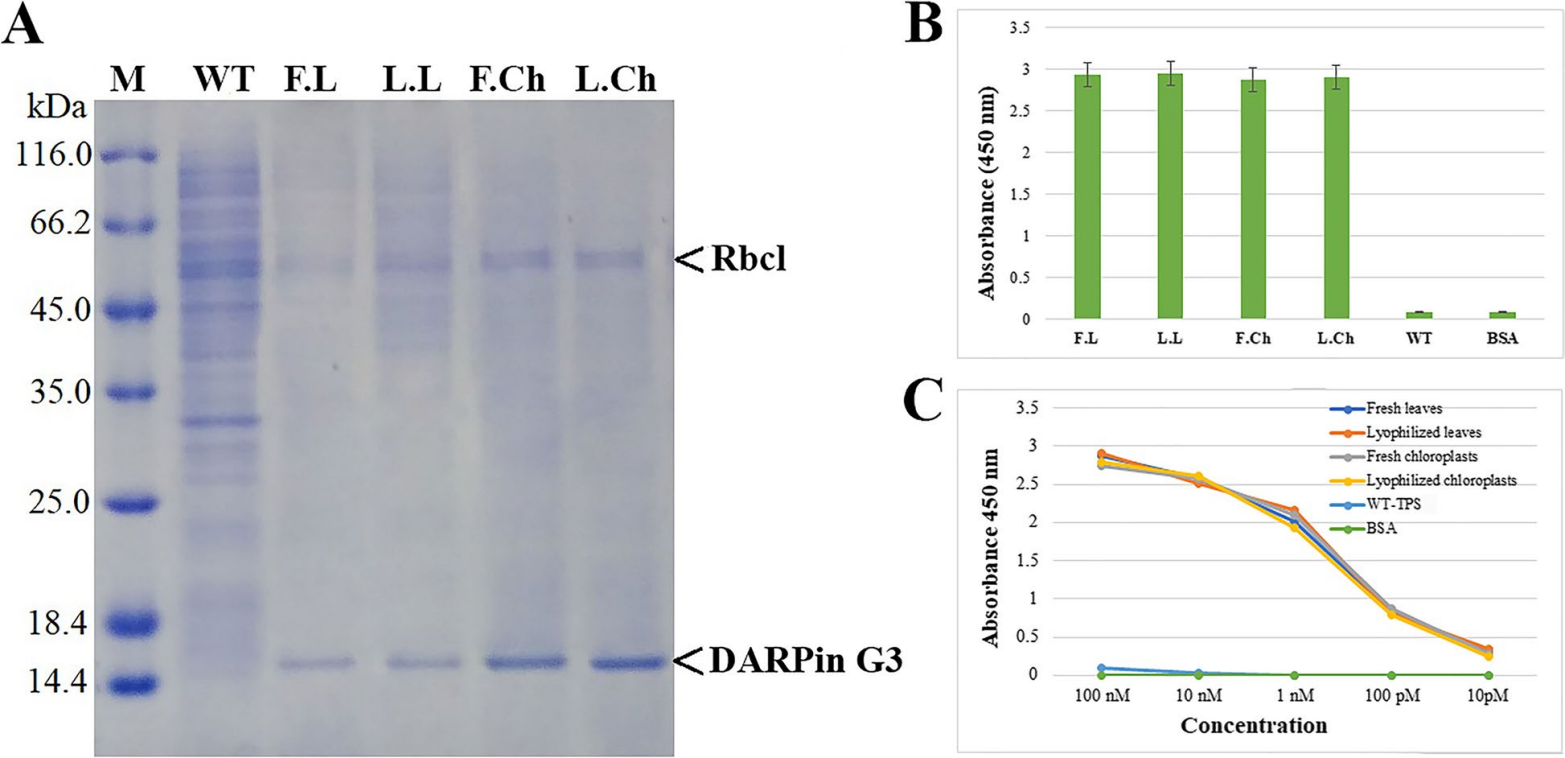
**A** SDS-PAGE analysis of chloroplast-made DARPin G3 purified from fresh leaves (F.L), lyophilized leaves (L.L), fresh chloroplasts (F.Ch) and lyophilized chloroplasts (L.Ch) of transplastomic tobacco plants. TSP extracted from a mature leaf of wild-type (WT) was also loaded as a control. On the margins, the positions of molecular weight markers (Broad Range Protein Molecular Weight Markers), DARPin G3, and RbcL are indicated. **B** HER2-ECD binding activity and (**C**) affinity of chloroplast-made DARPin G3 purified from fresh and lyophilized leaves and chloroplasts of transplastomic tobacco plants. The total soluble protein of the wild-type (WT) tobacco plant and the bovine serum albumin (BSA, 1%, w/v) were used as negative controls. Data are the means ± SD of three independent experiments. Error bars represent the standard deviation of the mean

When DARPin G3 is used as an injectable protein, the purification of chloroplast-made DARPin G3 protein is necessary. For this purpose, a histidine-tag was incorporated into the chloroplastic coding sequence of the DARPin G3-expressing cassette. The chloroplast-made DARPin G3 was partially purified from the total soluble protein of fresh and lyophilized materials with the Ni-NTA Purification System. The Coomassie blue-stained SDS-PAGE of purified protein showed a 15 kDa band corresponding to the DARPin G3 protein. SDS-PAGE analysis of purified protein when an equal volume of purified protein was loaded showed that the fresh and lyophilized chloroplasts contained the highest amount of DARPin G3 (Fig. 3A). The 55 kDa band corresponds to the large subunit of Rubisco encoded by the chloroplast *rbcL* gene, which is the most abundant protein in nature. It requires further purification steps to be removed from the partially purified protein. The purified DARPin G3 was used for the binding assay to the HER2 extracellular domain and to HER2 on the cancer cell surface.

### Binding affinity of chloroplast-made DARPin G3 to the HER2 receptor

To evaluate whether the DARPin G3 protein purified from lyophilized leaves and chloroplasts of transplastomic tobacco plants preserved its biological function of binding to the HER2 receptor, we performed a HER2-binding ELISA assay. Purified DARPin G3 from lyophilized materials showed strong binding affinity to HER2. As shown in Fig. 3B, binding activity in ELISA did not show detectable differences among chloroplast-made DARPin G3 purified from lyophilized materials compared to those purified from fresh leaf and chloroplasts of transplastomic plants (Fig. 3B). The binding affinity test by ELISA using the purified chloroplast-made DARPin G3 from lyophilized leaves and chloroplasts exhibits a sub-nanomolar range of affinity for HER2 (Fig. 3C), which is comparable with DARPin G3 previously produced in *E. coli* [22] and *P. pastoris* [17]. The efficacy of binding to the HER2 receptor was retained after lyophilization and long-term storage for nine months at ambient temperature. Wild-type plants and BSA didn’t show binding to the HER2 receptor.

### HER2 specificity of chloroplast-made DARPin G3

After a binding affinity test, the ability of chloroplast-made DARPin G3 from lyophilized leaves and chloroplasts to bind to the HER2 receptor on the cell surface was assessed in vitro using flow cytometry and immunofluorescent microscopy. Two human breast adenocarcinoma cell lines with different levels of HER2 expression were used for these purposes. The SKBR-3 line has been shown to strongly overexpress HER2, and MDA-MB-231, which has not been shown to overexpress HER2.

**Fig. 4:**
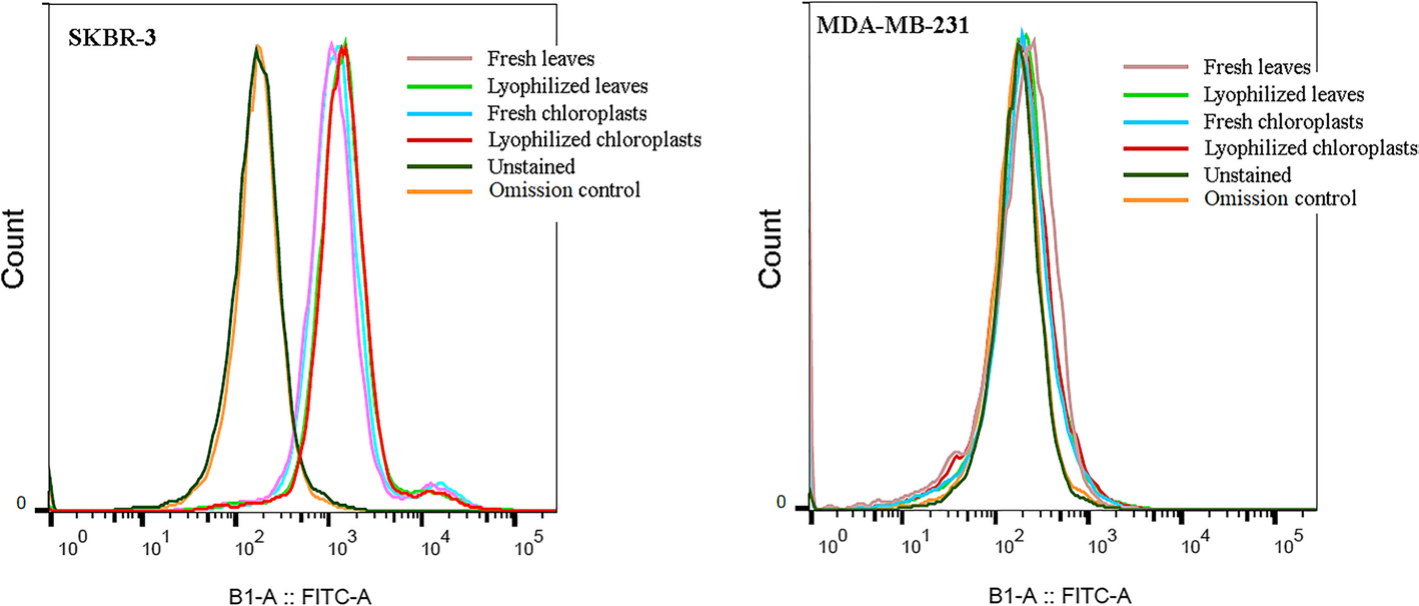
Characterization of HER2 binding of chloroplast-made DARPin G3 from fresh and lyophilized leaves and chloroplasts of tobacco transplastomic plants by flow cytometry in human HER2-positive (SKBR3) and human HER2-negative breast cancer cells (MDA-MB-231). Cells incubated with PBS or anti-His-tag antibody followed by FITC-conjugated antibody were used as unstained and omission controls, respectively. Data from representative experiments are shown

The flow cytometry results of the treated breast cancer cell lines demonstrated that chloroplast-made DARPin G3 from lyophilized leaves and chloroplasts bound to HER2-positive human breast cancer cells (SKBR-3), but did not bind to HER2-negative human breast adenocarcinoma cells (MDA-MB-231) (Fig. 4). Their performance was comparable with DARPin G3 purified from fresh leaves and chloroplasts and is in agreement with the formerly described DARPin G3 expressed in *E. coli* [20] or *P. pastoris* [17].

**Fig. 5:**
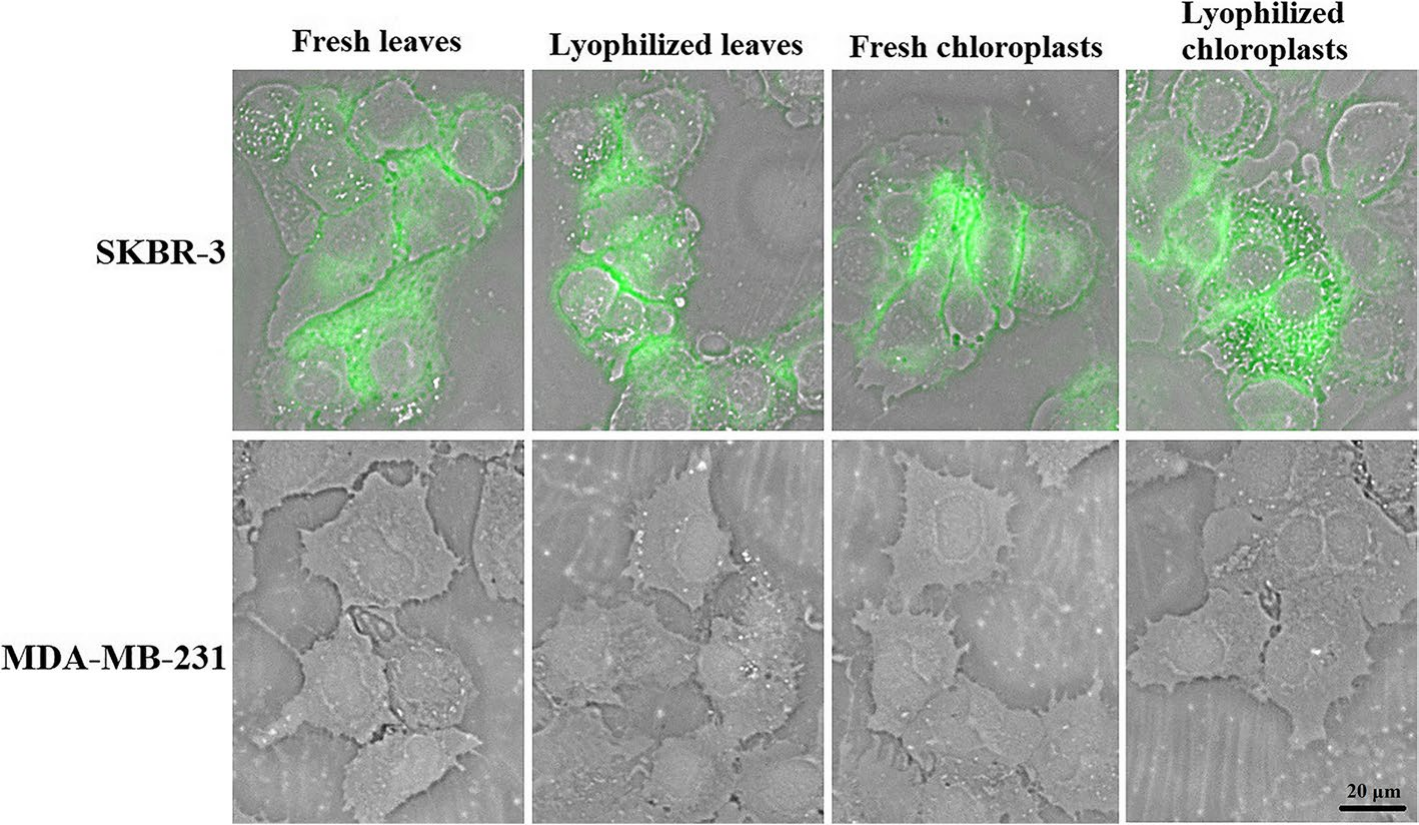
Binding of purified chloroplast-made DARPin G3 from fresh and lyophilized leaves and chloroplasts to HER2 on the SKBR-3 as HER2-positive and MDA-MB-231 as HER2-negative cell lines by immunofluorescent microscopy. Bound DARPin G3 was detected by incubating with an anti-His-tag antibody followed by a FITC-conjugated antibody, which showed HER2-specific targeting on the cell surface. Laser power and gain were kept constant, and brightness and contrast settings were adjusted equally. The scale bar represents 20 μm

Cellular binding of the chloroplast-made DARPin G3, purified from lyophilized leaves and chloroplasts, to HER2 on the cell surface was visualized by immunofluorescent microscopy using FITC tags. In contrast to MDA-MB-231 cells, we found a significant signal of FITC staining throughout the cell membrane in SKBR-3 cells (Fig. 5). The control experiment, which did not include the DARPin G3 or FITC-conjugated antibody, revealed no FITC staining at all. Some intracellular signals suggest that HER2 internalization has resulted in the internalization of at least a fraction of DARPin G3 proteins [23–25].

Retention of full activity and function of chloroplast-made DARPin G3, demonstrated by flow cytometry and immunofluorescent microscopy analysis, confirmed that once tobacco leaves and chloroplasts are lyophilized, DARPin G3 can be stabilized in plant cells at room temperature for over nine months.

## Discussion

Chloroplast transformation provides an opportunity for accurate incorporation of the target gene(s) into a predetermined region of the plastid genome through the mechanism of double homologous recombination. The chloroplast’s ability to correct protein folding and the formation of disulfide bridges, as well as its prokaryotic nature, are important features for the production of recombinant proteins through transplastomic strategies. The stability of expressed proteins is a major factor influencing the expression system’s overall viability. For proteins that do not require glycosylation, the chloroplast expression system is ideal for increasing protein yield, accumulation, and stability [26, 27]. Transplastomic plants could thus lead to a significant breakthrough in biotechnology, ranging from medication development to the large-scale commercial synthesis of enzymes and biofuels. As summarized in several reviews, chloroplast transformation is expected to offer unique advantages in the advancement of molecular farming for the production of biopharmaceuticals, vaccines, antibiotics, anti-microbial peptides, phytoremediation, industrial enzymes, biofuels, biomaterials [28, 29], and more recently, scaffold proteins as antibody mimetics [19]. Several functional therapeutic proteins have been expressed in tobacco chloroplasts in quantities comparable to or greater than those found in animal or bacterial bioreactors [30, 31]. Prolonged storage at ambient temperature, elimination of the cold chain, and purification steps provide the best opportunities for advancing low-cost plant-made pharmaceutical proteins.

Lyophilization is the most common method for preserving pharmaceutical proteins and vaccines in a dry form, which simplifies their storage and transportation without the arduous and costly cold chain. Extending this method to preserve natural biomaterials and cells in dry form would provide similar advantages. In comparison to current production methods, the use of this system in plant cells producing recombinant proteins has several cost-saving advantages in terms of production, purification, storage, and transportation. Additionally, the lyophilization process, which removes water from plant cells, enhances the concentration of protein drugs based on weight. As well as the foreign protein’s stability and functionality, the ability to deliver an appropriate dosage and consistency will be critical to the success of using freeze-dried material. Several studies in recent years have reported that lyophilization of transplastomic leaves expressing biopharmaceuticals or vaccine antigens can preserve them in a highly stable form, reduce costs, and facilitate storage, processing, and purification, resulting in simpler and more cost-effective immunization protocols [32–37].

Cancer is one of the most devastating diseases in the world, and breast cancer is the most common and leading cause of cancer death in women worldwide. Breast cancer with high levels of human epidermal growth factor receptor type 2 (HER2) expression, which accounts for 15–20% of all cases, has been associated with increased tumor aggressiveness as well as a high risk of recurrence and death [38]. Thus, information about HER2 expression levels is required for every invasive primary or recurrent breast cancer because it is critical for deciding whether to use HER2-targeting therapies or not [39]. DARPin G3 binds to HER2 with an affinity of 90 pM and exquisite selectivity [40]. According to preclinical and clinical studies, DARPin G3 is suitable for HER2 imaging because of its high-contrast imaging probe for several hours after injection [41, 42].

The selection of an appropriate expression system is influenced by the purpose, function, productivity, bioactivity, and physicochemical properties of the target protein [43]. DARPin G3 has previously been produced in *Escherichia coli* with a high *yield* in the range of 100–200 mg/L [20] and about 60 mg/L in *Pichia pastoris* [17]. However, recombinant protein production in both *E. coli* and *P. pastoris* expression systems faces its own challenges. For most cases, the formation of inclusion bodies in bacterial hosts, the extraction and purification of recombinant proteins to nontoxic levels, and the removal of highly stable endotoxins from bacterially produced biopharmaceuticals pose a major challenge for the large-scale recovery of bioactive proteins [44, 45]. In yeasts, low transformation efficiency, identification of high expression strains, improving secretion efficiency, and decreasing hyperglycosylation are the leading challenges when producing recombinant proteins [46, 47].

We recently reported the high-level expression of DARPin G3 (about 3.4 mg/g leaf fresh weight) in the chloroplast of tobacco plants as the first antibody mimetic produced in a plant expression system. Its functionality in HER2 imaging is comparable to that of previously produced DARPin G3 in *E. coli* and *P. pastoris* [19]. In this study, we investigate the long-term storage effect (for nine months at 22–24 °C) on the stability and functionality of chloroplast-made DARPin G3 in lyophilized leaves and isolated chloroplasts of transplastomic tobacco plants. Leaves and isolated chloroplasts from fully grown homoplasmic transplastomic tobacco plants were freeze-dried, lyophilized, and stored at room temperature for nine months. Western blot and ELISA analyses of lyophilized materials were performed to confirm the accumulation and quantification of chloroplast-made DARPin G3 in lyophilized materials. The detected band patterns using Western blots between protein samples were identical in fresh and lyophilized materials. Our results demonstrated that the chloroplast-made DARPin G3 from lyophilized leaves and chloroplasts retains an expression profile comparable to that of fresh leaves and chloroplasts. The 32-fold diluted lyophilized chloroplast sample showed that there was a 32-fold increase in DARPin G3 content per gram of lyophilized chloroplasts when compared to fresh leaves. The functionality of the chloroplast-made DARPin G3 protein in lyophilized leaf materials was confirmed using flow cytometry and immunofluorescence microscopy. It has been demonstrated that chloroplast-made DARPin G3 can maintain its activity and binding affinity even after prolonged storage at room temperature. Our findings reaffirmed that the pharmaceutical protein stored in lyophilized chloroplasts as well as leaves can be preserved for a long time at ambient temperature without losing its efficacy.

The estimated costs of recombinant protein production and delivery in bacterial, insect, or mammalian cells are much higher when compared to plants [48]. The simplicity and unlimited scalability of protein production and the lack of microbial contamination can make plant-made recombinant proteins economical and safer for large-scale production [27, 49, 50]. Tobacco was chosen as an alternative expression system for expression of DARPin G3 because it is a leafy plant and has a high potential for producing more than 90 tons/ha of leaf biomass with a plant density of 20,000 plants per hectare over three leafy harvests [51]. For long-term storage, the isolated chloroplasts and harvested leaves could be lyophilized and stored in air-free, compressed packages with minimal space. The lyophilized leaves and chloroplasts lose approximately 93% and 48% of their fresh weight, respectively. Because the volume occupied by fresh and lyophilized leaves is much higher than that of fresh or lyophilized isolated chloroplasts, the isolated chloroplasts can be of particular interest as a base material for extracting recombinant proteins. Since the chloroplast content of the tobacco cells from the leaf blade in our work was about 6.38% and midribs consist of 40% of leaves, the fresh chloroplast yield will be approximately 3.42 tons per hectare, which after lyophilization will decrease to 1.68 tons per hectare. Our results indicated that approximately 111.3 mg of chloroplast-made DARPin G3 can be obtained per gram of lyophilized chloroplasts. Accordingly, a total of 187 kg of purified DARPin G3 can be produced per hectare over 90 to 120 days from transplanting to the last harvest of leaves. This corresponds to nearly 1.2- million-liter E. coli cultures grown in at least one fermenter with a 10,000- to 13,000-liter tank for 90 to 120 days.

## Conclusion

In this project, we demonstrate that the chloroplast transformation system and chloroplastic encapsulation within lyophilized materials would be cost-effective for the production of functional DARPin G3 for HER2 imaging. Our results provide the basis for the expression of other scaffold proteins and indicate that lyophilization of transplastomic leaves and chloroplasts expressing scaffold proteins, without the need for additional lyoprotective components, can further lower costs and simplify downstream processing, purification, and storage.

## Materials and methods

### Plant materials

**Fig. 6:**
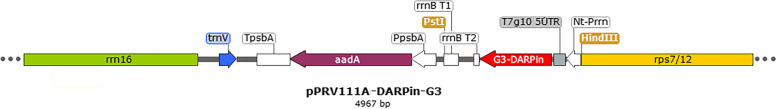
Physical map of fine structure for the chloroplast-specific DARPin G3 expression cassette, Nt-Prrn: ribosomal RNA operon promoter from tobacco; T7g10 5´ UTR: 5´ untranslated region of bacteriophage T7 gene 10; DARPin G3: coding sequence of DARPin G3, TrrnB: rrnB 3´ untranslated region from *E. coli;* PpsbA: promoter and 5´ UTR of the *psbA* gene; *aadA*: aminoglycoside 3´- adenylytransferase gene; TpsbA: terminator of the *psbA* gene

Tobacco (*Nicotiana tabacum* Cv. Perega) plants harbor pPRV::DARPin G3 constructs expressing the chloroplast-made DARPin G3 used in this study were obtained from our previous study (Fig. 6) [19]. Briefly, the DARPin G3 expression cassette was constructed by fusing the codon-optimized coding region of DARPin G3 with the strong rRNA operon promoter (Prrn) and a T7g10 5´UTR-derived leader sequence at the 5´-end and the *rrn*B 3´UTR from *E. coli* at the 3´-end. The 5´UTR sequence contained a strong ribosome binding site (TAAGGAGTG), an epsilon motif as an enhancer sequence (TTAACTTTA) and a 10-nucleotide poly-A-spacer between the epsilon motif and the ribosome binding site. To facilitate the purification of the protein, the histidine-glutamate (HE)_3_-tag sequence was embedded at the 5´-end of the DARPin G3 encoding sequence. The final DARPin G3 encoding cassette with translation control elements was subcloned into pPRV111A [52]. The transformation was performed using the particle bombardment method on tobacco leaf explants and the regeneration of transplastomic plants in the selection medium containing 500 mg/l streptomycin [53]. Homoplasmic shoots were regenerated three times under stringent spectinomycin/streptomycin selection pressure. The preliminary confirmation of transgenesis and homoplasmic status of transplastomic plants was confirmed by polymerase chain reaction and Southern blot analysis, respectively. After three rounds of selection, inheritance assays of transplastomic seeds displayed a homogeneous population of antibiotic-resistant seedlings, confirming that they are homoplasmic [54].

### Isolation of chloroplasts

To prepare chloroplast proteins for downstream analysis, chloroplasts were separated from mature, green, and fully grown leaves from the mid-section of DARPin G3 transplastomic and wild-type tobacco plants, which were kept in the dark for 48 h to destarch the plastids. The leaves of transplastomic and wild-type plants, after removing the midrib, were finely ground and homogenized with 3 volumes (v/w) of ice-cold chloroplast isolation buffer (50 mM Tris-HCl, 0.35 M mannitol, 5 mM disodium EDTA, 0.1% BSA (w/v), and 1.0 mM 2-mercaptoethanol) using a motor-driven blender. The homogenate was centrifuged at 4 °C for 20 min at 1000 × g to pellet cell debris and nuclei after being passed through three layers of Miracloth. To isolate chloroplasts, the supernatant was decanted into fresh tubes and centrifuged at 4 °C for 20 min at 2500 × g. After discarding the supernatant, the green pellet was washed twice in the isolation buffer before final centrifugation for 10 min at 1500 × g. The supernatant was discarded, and the chloroplast pellet was utilized, either to extract total soluble proteins or by lyophilization.

### Lyophilization

Fully expanded leaves from transplastomic and wild-type tobacco plants expressing DARPin G3, after excision of the midrib, were sliced into small pieces measuring roughly 1 cm^2^, frozen in liquid nitrogen, and then lyophilized in a Christ Alpha 1–2 LDplus Freeze Dry System in a vacuum (0.036 mBar) at -55 °C for 72 h. Lyophilization of isolated chloroplasts was performed in vacuum (0.036 mBar) at -50 °C for 72 h. The lyophilized materials were stored at room temperature for nine months and were used for the protein extraction after being ground in a grinder for 2 min at maximum speed three times (each time, pulse on for 15 s and off for 30 s). In the case of lyophilized leaves, the ground materials were subjected to sieving (mesh size: 100).

### Protein immunoblot analysis

Total soluble protein was extracted from fresh and lyophilized leaves and chloroplasts of transplastomic and wild-type tobacco plants. 100 mg of fresh, fully expanded leaves grounded in the presence of liquid nitrogen or 100 mg of isolated chloroplasts were combined with 500 µl of cold extraction buffer (PBS 1X, pH 7.4, 150 mM NaCl, and 1X EDTA-free protease inhibitor), vigorously vortexed, and incubated for 30 min at 4 °C. Due to the weight reduction during freeze-drying (93% for leaves and 48% for chloroplast pellets), Lyophilized leaf tissue and chloroplasts were normalized with fresh leaf tissue and fresh isolated chloroplast weight, so that 7 mg of lyophilized leaves and 52 mg of lyophilized chloroplasts were used by adding 500 µl of extraction buffer and vortexed for 1 h at 4 °C for rehydration. The crude extracts were centrifuged at 13,000 rpm at 4 °C for 10 min to pellet cell debris. Supernatants were collected, and total protein concentrations were quantified by the Bradford protein assay (Bradford, 1976) using bovine serum albumin (BSA; Sigma Aldrich) as a standard.

As the normalized amount of fresh and lyophilized leaves and chloroplasts was used for protein extraction, we loaded gels on the basis of an equal amount of protein extract for Western blotting. About 50 µg of total soluble protein from each sample was boiled in sample buffer and separated by a 12% sodium dodecyl sulfate–polyacrylamide gel electrophoresis (SDS-PAGE) in reducing conditions. Separated proteins were electro–transferred onto a nitrocellulose membrane (Bio-Rad) using Mini TransBlot (Bio-Rad), following the manufacturer’s instructions. Chloroplast-made DARPin G3 was detected using rabbit anti-His-tag antibody as a primary antibody and goat anti-rabbit conjugated with horseradish peroxidase (HRP) antibody as a secondary antibody. The bands were detected with the addition of a DAB-peroxidase substrate solution.

### ELISA quantification of chloroplast-made DARPin G3 proteins

An enzyme-linked immunosorbent assay (ELISA) was used to measure the amounts of chloroplast-made DARPin G3 expression in fresh and lyophilized leaves and fresh and lyophilized chloroplasts of transplastomic and wild-type tobacco plants. About 50 µg of total soluble proteins in extraction buffer (PBS 1X, pH 7.4, 150 mM NaCl, and 1X EDTA-free protease inhibitor) from fresh and lyophilized materials were bound to a 96-well polyvinyl chloride microtiter plate overnight at 4 °C. Background was blocked for two hours at 37 °C with 300 µl of blocking buffer (1% BSA (w/v) in 1X PBS buffer containing 0.1% Tween 20 (w/v)). Wells were incubated for 2 h at 37 °C with rabbit anti-His-tag antibody diluted to 1:1000 with blocking buffer as the primary antibody, and then three times washed with PBS-T (PBS buffer containing 0.1% Tween 20 (w/v)). The blots were then incubated with a goat anti-rabbit antibody conjugated with HRP in blocking buffer at a dilution of 1:10000 as a secondary antibody for 2 h at 37 °C. The plate was washed again as stated above and developed using the 3, 3, 5, 5-tetramethylbenzidine (TMB) peroxidase substrate solution (200 mM citrate buffer, pH 3.95, 1% TMB, 0.01% H_2_O_2_). The reaction was terminated with 100 µl of 2 M H_2_SO_4_, and the optical density of each well was measured using an ELISA reader at 450 nm. For a standard curve, purified 15 kDa His-tagged standard protein was added in a serial dilution ranging from 3 to 25 ng/ml to the microplate and processed as described above. The standard curve was used to quantify the amount of DARPin G3 protein in the total soluble proteins that remained in freeze-dried materials compared to fresh tissue. The yield of DARPin G3 was calculated and expressed as a percentage of total soluble protein (TSP) and as the amount of DARPin G3 protein (mg/g).

### Affinity purification of chloroplast-made DARPin G3

The chloroplast-made DARPin G3 was purified directly from total soluble protein extracted from fresh and lyophilized leaves and chloroplasts of transplastomic tobacco plants using the QIAexpress Ni-NTA Protein Purification System (QIAGEN). Total soluble proteins were mixed with 4x binding buffer (2 M NaCl and 2X PBS, no imidazole) at a 4:1 ratio in batch mode and incubated for 2 h at 4 °C. For the washing and elution steps, the protein–resin complex was packed into a column. The column was then washed with the wash buffer (0.5 M NaCl and 0.5X PBS, no imidazole) and eluted in 0.5 mL fractions for three times with the elution buffer (0.5 M NaCl and 0.5X PBS containing 200 mM imidazole). The eluted fractions were dialyzed against PBS three times, aliquoted, and stored at − 20 °C. SDS-PAGE and Coomassie blue staining were performed to detect chloroplast-made DARPin G3.

### HER2 receptor binding assay

A binding test was carried out according to our previously described method [19] to measure the affinity of chloroplast-made DARPin G3 from lyophilized materials of transplastomic tobacco plants for the extracellular domain (ECD) of HER2 in 96-well pre-coated plates with the HER2-ECD. 100 µL of 1 g/mL HER2-ECD (Sino Biological, 10,004-HCCH) were used for the coating, which was then kept at 4 °C overnight. After being washed twice with phosphate-buffered saline solution containing 0.1% tween-20 (PBS-T), the plate was blocked of non-specific binding sites on a shaker for 1 h at room temperature with PBS-T containing 1% BSA. The ELISA procedure used a serial dilution ranging from 10 to 100 nM of purified chloroplast-made DARPin G3 in PBS-T/BSA, which was applied to triplicate wells in 100 µL volumes and incubated at room temperature for 1 h with shaking. After that, each well was washed three times with 200 µL of PBS-T. A rabbit anti-His-tag antibody (1:1000 in PBS-T/BSA), which recognizes the N-terminal histidine-glutamate (HE)_3_-tag of the chloroplast-made DARPin G3, was used to probe the binding and was incubated for an hour at room temperature on a shaker. After three washes with PBS-T, the goat anti-rabbit antibody conjugated with HRP was incubated for one hour at room temperature on a shaker in a final volume of 100 µL of PBS-T/BSA at a dilution of 1:10000. Following this, each well was washed with 200 µL of PBS-T for triplets. 100 µL of TMB solution was used to create the ELISA, which was then incubated at room temperature until a satisfactory colorimetric shift was seen. The reaction was stopped by adding 100 µL of 2 N H_2_SO_4_, and absorbance readings were measured at 450 nm using an ELISA plate reader. A total soluble protein of wild-type plants and bovine serum albumin (BSA) served as negative controls.

### Cell culture conditions

We examined the HER2 specificity of chloroplast-made DARPin G3 from fresh and lyophilized leaves and chloroplasts using two distinct breast cancer cell lines that express HER2 to varying extents. SKBR-3 cells, a highly expressed HER2 breast cancer cell line, and MDA-MB-231 cells, which hardly express HER2, were provided by the Immunology Research Center (Tabriz, Iran). SKBR-3 and MDA-MB-231 cells were cultured in cell culture flasks containing RPMI 1640 medium supplemented with 1% penicillin (10,000 units/ml), 1% streptomycin (10 mg/ml), and 10% fetal bovine serum (FBS) at 37 °C in a humidified incubator with 5% CO_2_.

### Flow cytometry

The binding specificity of the chloroplast-made DARPin G3 from fresh and lyophilized plant materials to HER2 on breast carcinoma cell lines was examined via flow cytometry by following the procedures as described by us [19]. In brief, two distinct cancer cell lines were individually prepared. After removing the medium, the cells were treated for 10 min with 5 ml of 0.2% EDTA. The cells were then transferred to tubes and centrifuged for 5 min at 4 °C at 1000 rpm. After removing the EDTA-containing supernatant, 5 ml of fresh medium was added. 1 ml of cells counted and diluted to 10^6^ cells per ml were used for each test condition. After washing the cells with cold PBS containing 1% BSA, 100 nM chloroplast-derived DARPin G3 was added to the cells and incubated at 4 °C for 1 h. Following a cold PBS/BSA wash, cells were incubated for 1 h at 4 °C with 200 µl of rabbit anti-His-tag antibody diluted in PBS/BSA (1:1000). The cells were then washed with cold PBS/BSA, followed by incubation for 30 min at 4 °C in the presence of 200 µl of fluorescein isothiocyanate-coupled donkey (FITC) anti-rabbit antibody at a dilution of 1:1000. After washing with cold PBS, the cells were resuspended in 500 µl of cold PBS and analyzed on a MACSQuant 10 Flow Cytometer (Miltenyi Biotec, Germany) at a flow rate of 500 s^−1^. Fluorescence was detected at 525 nm after being excited with an argon laser at 488 nm. Cells were then gated according to size scatter, forward scatter, and pulse width, so only single cells were analyzed. Negative groups included cells that had not been treated or that had been treated with anti-His tag antibody followed by FITC donkey anti-rabbit IgG. A total of 10,000 cell events were recorded per sample. Fluorescence data was analyzed using the software FlowJo (Tree Star, Ashland, OR). Cells treated with PBS or antibodies in the absence of chloroplast-made DARPin G3 were used as controls.

### Immunofluorescent microscopy

To begin, cells were seeded into sterile 96-well culture plates at a density of 1.0 × 10^4^ in RPMI 1640 growth media and incubated at 37 °C and 5% CO2 for the duration of the night. After removing the medium, the cells were washed three times with ice-cold PBS containing 1% BSA. Cells were treated for 1 h at 4 °C with 200 nM chloroplast-made DARPin G3 in PBS/BSA. Following three washes with cold PBS/BSA, the cells were incubated for 1 h at 4 °C with rabbit anti-His-tag antibody (1:1000 diluted in PBS/BSA) as the primary antibody. Cells were washed and incubated for 30 min at 4 °C in the dark with FITC donkey anti-rabbit IgG (1:1000 diluted in PBS/BSA) as a secondary antibody. After three washes with cold PBS, cells were analyzed using the Citation 5 Cell Imaging Multimode Reader (BioTek, Winooski, VT). The laser at 469 nm excited the DARPin G3-FITC, and the fluorescence of FITC was registered at 525 nm.

## Data Availability

The datasets used and/or analyzed during the current study are available from the corresponding author on reasonable request.

## Abbreviations

BSA: Bovine serum albumin
DARPin: Designed Ankyrin Repeat Protein
ELISA: Enzyme-linked immunosorbent assay
FITC: Fluorescein isothiocyanate-coupled
HER2: Human epidermal growth factor receptor 2
HRP: Horseradish peroxidase
PBS: Phosphate Buffered Saline
SDS-PAGE: Sodium dodecyl sulphate–polyacrylamide gel electrophoresis
TMB: 3,3',5,5'-Tetramethylbenzidine
